# The Healing Power of Play: Therapeutic Work with Chronically Neglected and Abused Children

**DOI:** 10.3390/children1030474

**Published:** 2014-12-09

**Authors:** Fraser Brown

**Affiliations:** Leeds Beckett University, City Campus, Leeds LS1 3HE, UK; E-Mail: F.Brown@leedsbeckett.ac.uk

**Keywords:** play, playwork, child development, therapy, neglect, abuse, Romania

## Abstract

This article concerns a therapeutic intervention with a group of abandoned children living in a Romanian pediatric hospital. The children, ranging in age from one to ten years old, had suffered chronic neglect and abuse. They had previously spent most of their lives tied in the same cot in the same hospital ward. They were poorly fed and their nappies were rarely changed. Although able to see and hear the other abused children, they experienced little in the way of social interaction. The article focuses on the play-based methods that were employed to aid the children’s recovery, while at the same time highlighting the general benefits of this very specific therapeutic approach to children’s recovery and development. In particular, there is an exploration of concepts such as symbolic representation, negative capability, joining, and the significance of play cues. However, despite the clear value of these individually focused techniques, the article proposes the tentative hypothesis that the most powerful healing factor was the unfettered playful interaction between the children themselves. In other words, the children in a very real sense may have healed each other while playing.

## 1. Introduction

In the early 1990s, following the overthrow of the Romanian President, Nicolae Ceausescu, (see Deletant [1]) the Western media began to gain access to the former communist state of Romania. They discovered a country whose institutions were largely failing to cope, partly because of the collapse of its financial systems, but substantially because of the legacy of the Ceausescu era. This was one of the poorest countries in Europe, within which lived around two million Roma people—the poorest of the poor, making up around 10% of the population. Ceausescu’s people had been subjected to a raft of bizarre dictums issued by the President. For example, in order to build up the country’s industrial base, he had decided there was a need to increase the size of the population. Therefore, contraception was banned for families with fewer than five children, and women were medically “examined” by a special branch of the police to make sure they weren’t having abortions [2]. As a result of these and other factors, more than a hundred thousand children were living in orphanages [3]. Many were HIV+, and within days of moving over to full-blown AIDS, they would die. For a short while, the Western media was full of images of dying babies, and the outpouring of charitable aid was enormous. However, interest gradually waned, and by the end of the century there was a widespread assumption that the issue had been addressed. It had not.

This article concerns the impact of a therapeutic playwork project on a group of sixteen abandoned children living in a ward of a Romanian pediatric hospital, ten years after the overthrow of Ceausescu. The project was the subject of a research study that focused on the children’s development [4]. The article draws on extracts from a research diary kept by Sophie Webb during the early months of the project. A far more extensive version of that diary appears as a chapter in *Play and Playwork: 101 Stories of Children Playing* [5]. All the children’s names have been changed in order to preserve their anonymity.

The children, ranging in age from one to ten years old, had suffered chronic neglect and abuse. They had spent most of their lives tied in a cot; they were poorly fed and their nappies were rarely changed. Although able to see and hear the other abused children, they were unable to leave their cots, and so experienced little in the way of social interaction. Some of the children were from the nearby Roma communities, and it emerged as the project proceeded that some of them had varying degrees of undiagnosed disabilities. The focus of our study was the children’s play development, which we assessed using an instrument developed for an earlier study [6]. During a period when nothing changed in their lives, other than their introduction to the playwork project, the children themselves changed dramatically. Their social interaction became more complex; physical activity showed a distinct move from gross to fine motor skills; the children’s understanding of the world around them was improved; and they began to play in highly creative ways. They no longer sat rocking, staring vacantly into space. Instead they became fully engaged active human beings. Our conclusion was simple, but striking: The children’s developmental progress was clearly identifiable, and apparently made possible through their experience of the therapeutic playwork project.

The therapeutic playwork project began in the summer of 1999 and continues today, albeit in a much changed form. It started as a result of the concern of the newly appointed Director of the Hospitals, Cornel Puskas. Although he was neither a pediatrician nor a psychologist, when confronted with a ward full of disturbed children sitting rocking in their own solitary worlds, he was reminded of one of the most powerful conclusions from the studies of Suomi and Harlow [7]: “play is of utmost importance for the subsequent social well-being of the individual and those around him”. In common with most Romanian institutions at that time the hospital had no spare money. Therefore, hoping to help the children recover, he set aside a room to be used as a “playroom” and approached the UK charity *White Rose Initiative* [8] for funding to employ someone to play with the children. WRI employed Edit Bus as the first Romanian playworker, and brought her to Leeds Metropolitan University [9] for a specially designed training course run by myself. Upon her return to Romania, Edit worked with the children for four months by herself, before being joined by Sophie Webb (a Leeds Met. playwork student) for an extended period, and later by me for briefer periods. Towards the end of the first year, WRI expanded the staff team to four Romanian playworkers.

In the early days of the project, Edit and Sophie had to untie the children in the morning, bathe them, change their nappies and feed them properly, before taking them to the playroom. The two playworkers worked with the children all day, bathing, changing and feeding them as and when necessary, and enabling them to begin the long road to recovery through play. At the end of each day, the children were returned to their hospital ward. As soon as the playworkers left the hospital, the nurses went into the ward and tied the children to their cots for the night. This daily pattern continued for at least the first year of the project. No amount of pleading or persuasion could change the nurses’ behavior. I have often been asked for an explanation of this “inhumanity” on the part of the nurses. It is of course difficult to understand. Was it rooted in contempt for disabled children, or possibly discrimination towards Roma children? Both prejudices were widespread in Romanian society. Perhaps the nurses were busy and under-resourced, and found the children less troublesome when they were tied up. Possibly they did not see their role in terms of care, but rather more as wardens. Perhaps they were somehow influenced by the general lack of respect for the individual of the Ceausescu regime.

## 2. Catastrophic Implications for Development

The psychiatrist Stuart Brown [10] has shown that when children are deprived of play, the consequences can be catastrophic. The emotions of this group of children were in turmoil, although for much of the time it would be more accurate to describe their emotions as “on standby”. Before the project started they just stared vacantly into space, rocking to and fro in that rolling motion so familiar to anyone who witnessed the television images emanating from Romania in the early 1990s. Apart from the overpowering smell of urine and excrement, the most striking thing about entering the ward was the silence, an experience also highlighted more recently by Nathan Fox, Director of the Child Development Laboratory at the University of Maryland, in connection with the Bucharest Early Intervention Project [11]. There was none of the usual noises heard in a typical pediatric ward in the UK, where we might expect to hear a combination of crying, laughing, yelling, *etc.*

The children generally looked several years younger than their actual age. For example, we worked with a ten year old boy (complete with nappy), who will call Nicolae. He could easily have passed for a three year old toddler in any UK nursery. When first taken out of his cot, he just walked around the room from one cot to the next, all the time holding on to the bars. If we stood him in the middle of the room, he would simply drop down, crawl across the floor and pull himself up with the bars of the cot; after which he would continue walking round the room gripping the bars of the cots. This lack of confidence was fairly typical, and was exacerbated by the fact that the children’s gross motor skills were poorly developed compared to what Gallahue *et al*. [12] suggest would be considered the norm for their age. Those who tried to walk did so in a rather awkward toddler-like fashion, with the result that they often fell and hurt themselves. Their fine motor skills were virtually non-existent.

Although on the surface they appeared to be largely emotionless, the children also exhibited apparently irrational fears, which could pitch them without warning into frantic rocking. This unfortunately caused them to harm themselves, because the violent rocking motion meant their heads contacted the bars of the cot, or sometimes the wall outside the cot. There were also examples of self harm, as defined by the psychotherapist and social work author Steven Walker [13]. For example, one little girl who was punished by a nurse for crying when she had been given an injection, spent the following hour sitting in the cot, banging her head against the wall and scratching at herself. By the time the playworkers arrived the next morning she had damaged her arms badly enough to have drawn blood.

There was minimal meaningful social interaction. Despite living most of their lives in a hospital ward with fifteen other children, they had not formed any relationships with each other. Presumably the fact that their movement was so restricted meant they hardly had the chance to interact. Another socially limiting factor was the absence of any verbal exchanges between the nurses and the children. Thus, the children had no opportunity to form an attachment to a sensitive, consistent and emotionally warm carer. According to Bowlby [14] that would be problematic in terms of future relationship building.

The children exhibited little sense of individuality, a fact that was not helped by their hospital records which for example sometimes gave them the wrong name, and also gave many of them the same birthday. We eventually worked out that no-one had kept any records of these children until they were admitted to this particular ward. Their common “birthdays’ merely reflected the fact that they had been moved to this ward on the same day. In most cases we managed to find out their real names by virtue of the fact that this was a small town where everyone knew everyone else’s business. For example, a passing policeman was able to identify one of the children, because he knew the child had been born in prison.

The classic quote from the developmental psychologist Brian Sutton-Smith [15] sums up the powerful nature of the play experience, and at the same time touches on the awful consequences of a lack of play:

*“The opposite of play—If redefined in terms which stress its reinforcing optimism and excitement—Is not work, it is depression. Players come out of their ludic paradoxes ... with renewed belief in the worthwhileness of merely living.”*

The static nature of the children’s world meant they had no sense of fun and playfulness, and they initially showed few signs of cognitive functioning. In a remarkably short period of time, all that would change.

## 3. Early Developments

In the early days of the project it was hard to assess which children had been borne with a disability, and which were merely suffering from years of neglect and abuse. However, those distinctions quickly became apparent as the children began to develop. A child who we will call Virgil (a six year old in nappies, looking about two years old) went from silently rocking in his cot to meaningful social engagement, in the form of playful conversation, in the space of six months. He made dozens of new discoveries every day, and excitedly shared them with his new friends. For example, when he worked out how to switch on a torch he toured the playroom tapping his playmates on the shoulder and pointing at the wall where he was shining the beam of light. That single example contains elements of considerable social, physical and cognitive progress in just six months.

On the other hand Olivia, who had been born two months premature and weighing less than two pounds, clearly had serious learning difficulties. She spent much of her time inspecting the toys in great detail, but the next day she would be inspecting the same toys. When Virgil showed her his torch discovery she reacted with enthusiasm, but largely as a result of the social contact, rather than out of any real understanding of what he was showing her. Nevertheless, during the first year she began to walk independently, and was able to join in with a range of simple activities. Perhaps most encouraging was the fact she appeared to understand the complex idea that games have rules which apply to everyone.

Although every child made progress, some forged ahead at such a rapid rate that it has forced me to question my previous assumptions about attachment theory, the long term impact of abuse, and the “stages” view of child development suggested by developmental psychologists, such as Erikson [16] and Piaget [17], especially in light of the chaotic way in which development occurred. In fact, the change in the children was so remarkable that a colleague commented to me, “it is almost as if their intellect has been sitting there, waiting to be switched on”. Within the first eighteen months, thirteen of the original sixteen children were either adopted or fostered within Romania—Something that would have been extremely unlikely at the beginning of the project, but something that their changed demeanor and behavior almost certainly made possible.

Nevertheless, there were still signs of residual fear, and emotional insecurity, sometimes resulting in the sort of extreme regressive response originally conceptualized by Anna Freud [18]. For example, on one occasion the children were playing happily in their “salon”, when the hospital’s accountant appeared to tell one of the playworkers there had been a problem with her wages this week, and she would not get paid until Monday (instead of Friday as normal). A slightly tense discussion ensued between the playworker and the accountant, albeit at no time did it get heated or aggressive. I looked around the salon and every single child had climbed back into their cot and was sitting rocking! This led me to begin to formulate one of the conclusions summarized later in this article, *i.e.*, that it may not be possible to recover the emotional equilibrium of such children. However, in most other areas of development, the children made remarkable progress.

## 4. Some Significant Indicators of Recovery

There was substantial development in the social skills of all of the children. Within four weeks they all learned how to initiate contact, and generally responded appropriately to contact initiated by another child. This rapid change is illustrated by the following extracts from Sophie Webb’s research diary [19]. For example, in the first week we find a complete absence of social skills:

“When I observed the children in the playroom, they were unaware of each other, fixed on their own activities – barely communicating. Some just sat and seemed bewildered and vacant.”

[20]

However, by Day 22 she recorded the following:

“The way the children sit around the table has proved to be more than just eating together. Olympia started to feed Nicolae today, so they are really interacting with each other so much more. They seem to be enjoying the social event.”

[20]

All the children’s communication skills developed, some more rapidly than others. For example, just sixteen days after Sophie’s arrival she recorded two of the children having an imaginary “phone conversation”. Another child, whose development was less speedy, was nevertheless sparked into a communication exchange when the playworker repeated a noise that the child made when showing signs of being happy:

“Today I started to repeat the noises that Elena makes ‘waaaaoooo waaaaoooo’ and her reaction was amazing! The look on her face was just like someone had spoken her language. It felt like a little break through as you can rarely communicate with her. I started to repeat this noise back to her and she responded by instigating the sequence when she saw me, exploring my face and trying to decide where the noise was coming from. By making myself the play environment Elena was comfortable to allow herself the freedom to communicate and investigate.”

[20]

Their language skills took longer to develop. At first the children had no language—Except that a couple of them could say “Hiya”, which presumably they had learned from the various religious groups who visited the hospital from time to time (but never for more than a couple of hours of largely pointless cuddling). However, once the project had been running for a few months, some of the children began to use words to explain themselves and to express their feelings, for example:

“Virgil especially loved the Lego bricks and we helped him build a house, which is ‘casa’. He walked around the room saying ‘casa—Casa’ in his little voice and he was so proud of it.”

[20]

Once the project had been running for about 9 months, the children’s language skills had developed to such an extent that the UK playworkers were sometimes out of their depth compared to their Romanian colleagues. For example, Edit would be able to stop a child doing something dangerous, with a single word.

Sturrock and Else [20] introduced the idea that the appropriate interpretation of play cues is a key ability in any playworker’s skill-set. Indeed it has been suggested that the level of sophistication of a child’s play cues can be taken as one indicator of their developmental level [6]. All the children developed cueing behavior, even Nicolae. Despite his clear learning difficulties, I nevertheless recorded him playing a game with one of the playworkers where he was copying her arm and leg movements, and exclaiming as he did so. Between them, they developed a kind of sitting down dance routine. Later in the day he sat down with another playworker and tried to initiate the same game by cueing with his own arms and legs. When she didn’t respond, he became quite frustrated, but persevered, and eventually got her to understand and follow his lead, so that they could dance together.

The muscular-skeletal development of the children is perhaps the most remarkable and unexpected of all the indicators of progress. When the project started all the children appeared to have considerably stunted growth, and although they began to get exponentially stronger and more active, we expected that their actual height would be forever damaged by the nature of their early lives. However, that proved to be untrue. Perhaps the most extreme example would be that of Nicolae, whom we have already said was a ten year old child with the build of a three year old. In 2009 I visited a special center in Sighisoara for youngsters with learning difficulties, where I was amazed to find Nicolae, who was now taller than me, and considerably stronger. Clearly, it is not possible to link this specifically to the act of playing, but it certainly raises the possibility that we are genetically programmed at birth with an optimal height.

## 5. Therapeutic Method

Our basic therapeutic method was to offer the children an environment that was as close as possible to a standard playwork environment, but to apply specific therapeutic techniques to individual children, as and when the need arose. Else [21] has suggested that playwork is characterized by the “three frees”: Freedom from payment; freedom of movement; and freedom of choice. Obviously in this unusual setting there was no question of a financial charge. Nor were there any restrictions on the children’s choice of activity (other than the limitations imposed by the nature of the playroom and its contents). However, the children were plainly not free to come and go as they pleased. To allow them to do so, would have endangered their safety, so we had to accept that restriction and work within it, albeit I have argued elsewhere [6] that no play environment is ever completely free from externally imposed parameters. In this case, the parameters were particularly unusual and challenging. Nevertheless, at a fundamental level, the key features of our approach were not very different from those applied in most playwork settings. These features are summarized here in no particular order:

### 5.1. Removing Barriers to the Play Process

The playworkers had to untie the children every day, release them from their cots, bathe them, feed them properly, *etc.* Clearly, the children would not have been able to benefit fully from their play if their movement was restricted, or they felt dirty or hungry or in a state of emotional distress. The very first entry in Sophie Webb’s diary illustrates this point:

“Every room was full of children in cots, but it was so quiet. Even when we entered the room there was no sound from the children. They just looked at us. The smell of urine in every room was almost unbearable. The emptiness. Each room had just the cots with plastic mattresses. The children were dirty and wearing clothes that were too big for them. Some were wearing jumpers as trousers, and none of them were wearing shoes. There were rags around their waists, which I later found out were ripped up sheets tied, to keep the nappies in place. These rags were also used to tie the children to the cots. Most children were sitting rocking and others were standing up banging the sides of their cots against the walls. Giving the children a cuddle was strange as they either held on too tightly, or they remained stiff and unfeeling.”

[20]

### 5.2. Enriching the Play Environment

In essence enriching the play environment was about ensuring that the playroom had the potential to enable the children to be playful—In other words a place where children are able to engage in non-serious activities for enjoyment, satisfaction and fun [22]. An enriched play environment holds greater potential for child development, but for the playworker this is not about imposing an adult agenda on the play space—Rather the playworker adopts an holistic approach to development; one that respects the idea that children develop while they are playing. There are various ways of enriching the play environment—For example, by providing a large variety of artifacts and activities, but leaving the choice of what to do at any particular moment in the hands of the child.

### 5.3. The Portchmouth Principle and Nicholson’s Theory of Loose Parts

Nicholson’s theory of loose parts [23] holds that “in any environment both the degree of inventiveness and creativity, and the possibility of discovery, are directly proportional to the number and kind of variables in it”. Underpinning this idea is the Portchmouth Principle [24] which states, “It helps if someone, no matter how lightly, puts in our way the means of making use of what we find”. Taken together these two ideas imply that the playworker’s role is to provide the basic tools and materials to enable the children to play. There is no need to tell them what to do. The play environment contains its own inherent stimulation in such circumstances. For example:

“Whilst Alex was sitting in a big yellow box Virgil started to play a game with him, involving an imaginary object. He pretended to receive something from Edit and then took it back to Alex in the box, who took it from him and put it in his lap. The spontaneous interaction between them both was fascinating to watch. Afterwards Virgil continued playing with the yellow plastic box, by putting it on his head and walking around the room, which made me laugh and laugh. He created a sort of obstacle course out of the cots and tables.”

[20]

### 5.4. A Non-Interventionist Approach

For most children the only time when they are in control of their world is when they are playing. Therefore, if playworkers are to avoid “adulterating” that experience, they have to ensure that wherever possible they are following the child’s agenda [21]. This is especially important in a therapeutic setting, where according to Axline [25], children will find a solution to their problems in their play, so long as they are given sufficient time and space. In the case of the therapeutic playwork project in Romania, where the children required a stronger presence over a more extended period, it nevertheless remained the case that most of our interventions were a response to the specific play behaviors of each child.

### 5.5. Negative Capability

According to Fisher [26] it is sometimes by appearing to do nothing that the playworker achieves most. When we rush to intervene we limit our capability and the children’s creativity, but she says:

“By actively ‘being with’ a situation, without trying to change it, influence it, explain it or understand it, we keep all options open—Anything is possible and nothing is closed off. ... the play itself will do what it needs to do, but we need to watch the play carefully with this attitude of negative capability so that we really ‘know’ what is happening. We will then know intuitively, when or if an intervention is needed.”

Thus, a playworker needs to be open-minded, non-judgemental, and non-prejudiced, and by so doing s/he provides an environment that encourages imagination, creativity, exploration and experimentation—and hence development.

One day, during the hot summer months we took the children outside for a picnic. Everyone was given a cup of cold water. A little boy, who clearly exhibited learning difficulties, fell over on the rough ground and spilt his drink. He then approached the playworkers to ask for more water. They topped up his cup, whereupon he hurled the contents all over the playworkers.

It is likely that most adults in other professions would have chastised him, or at least given him some moral instruction about not being a naughty boy. However, the playworkers just became really excited, because they recognized real developmental progress in this one act. He had obviously learned from his accident that the water could be tossed from the cup. He had also worked out that he could play a trick on his playworkers. This was quite sophisticated reasoning and remarkably perceptive for a child who had been born with brain damage, and spent most of the subsequent ten years tied in a cot. His sense of achievement was clear, but might have been missed if the playworkers had been adopting a more typically adult (instructional) frame of mind. Indeed, if the playworkers had either chastised him or offered him guidance regarding “good” behavior, they would probably have dampened his enthusiasm to try things out.

### 5.6. Using Personal Life Skills

Sympathy, empathy, mimesis, affective attunement and the sensitive interpretation of play cues, are skills and abilities that are essential for a well-adjusted social being. Children develop these skills while they are playing. They are not skills that can be taught in a classroom. They are all skills that are essential to playworkers if they are not to misread the sort of situations that confront them every day [6]. This was abundantly clear while working in the hospital.

For example, on one occasion a playworker was engaged in a game of chase with one of the children. Although the child was chasing the playworker, he wanted the playworker to chase him. As they were running round the cots he began to issue little cues. First he banged a table with his hand, but the playworker missed the play cue she had just been given. Next, he knocked over a mattress, and clasped his hands to his face in mock horror. This was a more obvious cue, and yet it was also missed. At each missed cue the child was obliged to keep chasing the playworker, even though he didn’t want to do so—otherwise the game would have stopped. Finally, he ran past the playworker’s coat, which was hanging from a door handle. He put his hand into the coat pocket, pretending to steal something. At last the playworker got the message, and started to chase the child who yelled excitedly, quickly allowing himself to be caught. The pair ended up rolling around on the floor with the child giggling triumphantly.

The fact that the child quickly allowed himself to be caught, once the playworker started chasing him, provides a strong indication of his intended aim, and his new-found ability to reason things out. If the playworker had not been adopting a playful approach, she might have felt obliged to reprimand him for touching her coat, or for trying to steal something from the pocket. However, that would be reflective of an adult agenda, and would be interpreted as such by the child. Of course this is just one example, and it would be wrong to suggest a failure to respond to the child’s play cues is damaging on every occasion. However, repeatedly failing to understand and respond to the child’s real motives would be likely to have negative consequences in terms of that child’s development. At best they would become disheartened; at worst it is suggested by Sturrock and Else [21] that children are likely to develop neuroses.

### 5.7. Joining the Child’s Agenda

When we first worked with Nicolae he was obsessed with shoes. He spent most of the day trying to take people’s shoes off, or putting his shoes onto other people (not just the other children, but anyone who came into the room). He built up collections of shoes, and sometimes had shoes on his hands as well as his feet. In fact, his behavior had similarities to the stereotypic repetitive behavior commonly seen in children with autistic spectrum disorders [27]. However, in line with the approach of the Autism Treatment Center of America [28], we did not try to force him to move away from his obsessive behavior, but instead attempted to use that behavior as a means of initiating contact and building trust. The ATCA calls this approach “joining”. In playwork terminology, we were taking the child’s agenda as our starting point, and working with it [6].

What did this mean in practice? Sophie played “shoes” with Nicolae over and over again, until he began to interact with her. Very gradually a trusting relationship built up, which Sophie was then able to use to the benefit of Nicolae. As has already been mentioned he was reluctant to walk unaided. Several people had tried persuading him to let go of the bars of the cots and walk independently. However, once Sophie had formed a strong bond with him, she hoped the attraction of a hug from his playworker would over-ride his fear of falling. In the event she was correct. She was able to stand him in the middle of the room and he chose to walk towards her, instead of dropping to the floor and crawling across to the cots. These were the first independent steps of a child who had hitherto been tied to his cot and dismissed as “debil mintal” (mentally deficient), or even worse, “un imbecil”.

### 5.8. Creating Relationships and Building the Child’s Self-Esteem

One of the most significant elements of the preceding vignette is the way in which the relationship was developed with the child, and the importance of that in helping his development. As we have already seen the playwork approach has unique elements; most especially there is no attempt to take control of the child’s agenda. That is unusual for a child to experience, and often leads to the formation of a trusting bond with the playworker. Examples of this process may be seen in several of the vignettes already provided. The non-interventionist, non-judgmental and non-controlling approach carries with it a powerful message of respect for the child’s social, physical and emotional space [6]. Not only does this help the formation of a strong relationship, but it also helps to build the child’s self-esteem.

### 5.9. Cultural Competence

Regardless of the setting in which they are working, playworkers need to develop their own cultural awareness—Macro and micro. In other words they need to understand both the culture of the local community and also the children’s own sub-culture(s). Both will be characterized by their own unique “webs of significance”, *i.e.*, a complex intertwined mesh of social, political, economic, philosophical, spiritual and emotional principles that underpins everything that happens within any particular community. In this case, it was necessary for the playworkers to develop an understanding of several communities, *i.e.*, the country, the town, the hospital, the nurses, and as time went on the children’s own little community. All these things serve to create the “webs of significance” referred to by Geertz [29], and with which playworkers must aim to feel comfortable.

## 6. Longer Term Evidence of Progress

In an earlier article we reported on our research findings, analyzing the developmental changes identified in the children [30]. These were based on a “play value assessment guide” that focused on eleven distinct categories: Freedom; flexibility; social interaction and socialization; physical activity; intellectual stimulation; creativity and problem solving; emotional equilibrium; self-discovery; ethical stance; child-adult relationships; and general appeal, fun and enjoyment. In every category, for every child, the results showed some development, albeit there were substantial variations from one child to another. However, the findings showed that all the children underwent significant developmental change in the areas of social interaction and socialization, physical activity and general enjoyment of play. Before the input of the Therapeutic Playwork Project each child was isolated, physically weak, lacking in social skills, and generally showing signs of emotional distress. During the first year of the project the children developed a group identity; their gross and fine motor skills moved ahead at a rapid rate; they developed ways of communicating that enabled them to form friendships within the group; and they showed all the “normal” signs of happiness (smiling, laughter, readiness to take risks, the use of make-believe, *etc.*).

## 7. Six Years on

We have shown our video footage of the children’s progress during the first eighteen months of the project in all sorts of settings, and to a wide variety of audiences. The impact of these images remains as strong today as when the original filming took place. In many ways the material is timeless. Maltreatment of children occurs all over the world, albeit not usually in such an institutionalized way. However, the power of the images lies not only in the shocking consequences of neglect, but also in the idea that recovery is possible, even in the most adverse conditions imaginable. Not unreasonably a question we have often been asked is “where are they now, and have they continued to make progress?” Six years after our initial research [31] was presented I attempted to trace the subsequent lives of the original sixteen children.

Three of them were easy to locate, because they were still in the system having been transferred to a children’s mental hospital, where the conditions appeared to be extremely restrictive. We were given a permit to visit these children, but were not allowed past the gate-keeper’s hut. A nurse brought two of them to the hut to see us, and although there was some evidence of recognition, the children appeared to be in a dream, presumably because they had been given some form of calming “medicine”. When asked whether the children were given time to play out in the grounds of the hospital, the nurse just said “No!”.

The other thirteen children had either been fostered or adopted (all within Romania), which made them far harder to trace. In fact, it was only possible to locate seven of them.

One child, who we shall call Elena, had regressed substantially. I had previously visited Elena soon after she moved into her foster home, and she had formed a strong relationship with her new “grandma”. She seemed to be progressing well, and even sang me a nursery rhyme. Soon after that visit Elena was taken into hospital for some tests. While she was away her “grandma” died. Six years on, I found a child who had not spoken a word since coming out of hospital. It is not possible to identify the cause of this. Perhaps she had a bad experience in hospital; perhaps the trauma of losing her “grandma” was simply the last straw in a life of abuse. We shall probably never know.

Although it was not possible to conduct any formal testing, the other six children appeared to be progressing well. For example, one of them had been attending a German speaking nursery, and entertained himself by trying to teach me to count. He even showed himself to be a good mimic when he started to copy my poor attempt at speaking German. Another child was keen to talk about his toys. His foster parents insisted that he also demonstrate his reading skills and some basic arithmetic. I was introduced to several of the children at a special center that aims to help them catch up with their school friends. While talking to one of the workers, I felt a child tugging at my trousers, and a little voice said, “te cunosc” (I know you). Six years previously this was a child who Sophie had discovered tied to his cot, playing with his own feces—presumably for want of something to play with.

We cannot really know whether these seven children are representative of all thirteen adoptees. If they are, then the project appears to have been successful in the longer term on several counts:

- The children are all living in a supportive environment with caring families and a stable home life
- The children who were not born with some form of brain damage appear to have continued the progress made during their experience of the project
- Those children are generally behind in their schooling, but appear to be capable of catching up if given appropriate support
- Of the children who were born with some form of brain damage, all but one has continued to make progress, albeit the level of that progress varies considerably from one child to another
- Apart from Elena and the children who were in the mental hospital, all the children appear to have developed the skills necessary to make friends
- All the children have developed gross and fine motor skills, and appear to be physically healthy, even to the extent of having grown to full adult height
- All the children have developed a strong individual identity
- It was not possible to judge the emotional stability of the children, and in light of our experience during the project I do not feel confident about making any claims in that regard

## 8. Conclusions

Thankfully the hospital’s approach to the children changed dramatically after about eighteen months. Today all children are treated the same, no matter what their reason for being in hospital. They are bathed regularly, properly fed, and their developmental needs are addressed. Staff turnover saw some of the worst offenders move on, but I am convinced the major causal factor was the example provided by the WRI playworkers who were encouraged to treat the children with love and respect at all times.

In less than a year, these chronically abused and neglected children made the sort of progress that many experts assumed would be impossible. During the whole period of the research study when the children were not in the playroom with the playworkers, they were tied back into their cots by the nurses. They were not fed properly; they were not bathed; their nappies were not changed; and no-one gave them any meaningful attention at all. In other words, nothing changed in their lives during that first year except their experience of the playwork project. Therefore, it is sensible to ask what it is about playwork that contributed to the striking changes in the children.

Clearly the children’s learning and development resulted substantially from the playworkers’ ability to create an enriched play environment that was substantially supportive of the play process. The playworkers’ non-judgmental approach, coupled with a determination to take each child’s agenda as his/her own starting point, helped to create a good quality playwork environment—In other words, an environment that offered adaptability to the children, and so encouraged the compound flexibility process [31]. Through their empathy, and their ability to interpret the children’s play cues effectively, the playworkers were able to create strong trusting relationships, which in turn helps to enhance the children’s self-esteem [32].

If such approaches were applied in a typical playwork setting in the UK, we would take it for granted that children would learn and develop naturally. The remarkable thing about our experience in Romania was that this straightforward playwork approach appeared to work just as effectively with some of the most play-deprived children in the world. Given that the playworkers’ approach was generally non-interventionist, it is tempting to draw the conclusion that the major healing factor in all this was the children themselves. To substantiate such a claim would require more in the way of comparisons of different types of intervention, including play, to be able to isolate peer-to-peer play as the key causative factor. After all it is also possible that the sensitive interactive input from the playworkers might also have been a trigger and supportive factor in initiating a trajectory towards degrees of recovery. However, to conduct such a comparative experiment would be completely unethical. Therefore, all we can do is draw tentative conclusions, and propose cautious hypotheses. It is clear that the playworkers had an influence, but largely by virtue of the environment they created. It is, therefore, my hypothesis that the remarkable development we witnessed in such a short period of time was substantially stimulated by the children’s interaction with each other.
